# ‘Teach-back’ is a simple communication tool that improves disease knowledge in people with chronic hepatitis B – a pilot randomized controlled study

**DOI:** 10.1186/s12889-019-7658-4

**Published:** 2019-10-23

**Authors:** Sophie Tran, Gabrielle Bennett, Jacqui Richmond, Tin Nguyen, Marno Ryan, Thai Hong, Jessica Howell, Barbara Demediuk, Paul Desmond, Sally Bell, Alexander Thompson

**Affiliations:** 1 Department of Gastroenterology, St. Vincent’s Hospital Melbourne, Level 4 Daly Wing, 35 Victoria Parade, Fitzroy, Victoria 3065 Australia; 2 The University of Melbourne, Grattan Street, Parkville, Victoria 3010 Australia; 3 The Burnet Institute, Disease Elimination, 85 Commercial Road, Prahran, Melbourne, 3004 Australia

**Keywords:** Health literacy, HBV, Teach-back, Education, Communication, Liver, Cirrhosis, This study has been retrospectively registered with the Australian and New Zealand Trials Registry on 29th March 2019 - ACTRN12619000512123.

## Abstract

**Background:**

The low diagnosis rate and poor access to clinical care among people with CHB is a major barrier to reducing HBV-related morbidity and mortality in Australia. One explanation for this is a lack of disease-specific knowledge among people living with CHB. Health literacy has been shown to be important for maximising engagement with medical care and adherence to recommended management. The ‘teach-back’ communication strategy has been shown to improve patient understanding in other clinical areas. This study aims to assess disease-specific knowledge; and evaluate the efficacy of the teach-back strategy for improving HBV knowledge, compared to a standard medical consultation.

**Method:**

A randomized pilot study was conducted between February and June 2017. Participants were recruited from the liver clinic at an inner-city tertiary hospital. English-speaking patients aged ≥18 years and diagnosed with CHB were eligible for the study. Participants were randomised to a control group (medical specialist appointment) and intervention group (teach-back). Knowledge was assessed at baseline, immediately post-intervention and at one month using a validated questionnaire. Participants in the intervention group received a one-on-one teach-back session about CHB. The main outcome measure was a combined knowledge score of the domains assessed – transmission, natural history, epidemiology and prevention and clinical management.

**Results:**

Seventy participants were recruited (control *n* = 32, teach-back *n* = 38). Mean baseline knowledge score was 19.1 out of 23 with 55 (79%) participants scoring ≥17.3 (defined as high knowledge) (7). Sub-analysis of CHB knowledge domains identified focal deficits concerning transmission and whether HBV is curable. Knowledge scores were found to be positively associated with English proficiency and antiviral treatment experience (*p* < 0.05). Teach-back was associated with a significant increase in CHB knowledge at early recall (22.5 vs 18.7, *p* < 0.001) and at 1-month follow-up (21.9 vs 18.7, p < 0.001); there was no improvement in CHB knowledge associated with standard clinical consultant (early recall: 19.6 vs 19.4, *p* = 0.49, one-month follow-up: 19.5 vs 19.4, *p* = 0.94).

**Conclusion:**

In a tertiary hospital liver clinic population, baseline knowledge about CHB was good, but there were focal deficits concerning transmission and potential for cure. Teach-back was associated with improvement in CHB knowledge and it is a simple communication tool suitable for incorporation into a standard medical consultation.

## Background

Over 200,000 Australians were estimated to be living with chronic hepatitis B (CHB) in 2011, with higher rates in Aboriginal and Torres Strait Islander and culturally and linguistically diverse (CALD) communities [1]. CHB is a major cause of mortality; responsible for 53% of hepatocellular carcinomas (HCC) worldwide [2] and is increasingly recognised to cause significant morbidity and mortality within Australia where liver cancer is now the fastest growing case of cancer-related deaths [3]. A low diagnosis rate [1] and poor access to clinical care [4] among people living with CHB is a major barrier to reducing CHB-related morbidity and mortality. One explanation for this is poor disease-related knowledge among people living with CHB.

Health literacy (HL) is important for maximising engagement with medical care and adherence to recommended management [5, 6]. Studies assessing disease-related knowledge among Australians living with CHB have revealed gaps in patient understanding, particularly concerning modes of transmission, clinical management and consequences of CHB beyond the liver [7–9]. Despite current literature identifying knowledge gaps amongst patients with CHB, few studies have evaluated the effectiveness of an educational intervention in people already diagnosed with CHB. Educating people living with CHB could lead to improved coping mechanisms and empowerment to actively participate in their clinical management. Adherence to recommendations concerning prevention of transmission (family screening and vaccination, prevention of sexual transmission), monitoring (virological and HCC screening) and treatment (long-term antivirals) are particularly important to improving long-term outcomes of the individual and the community.

The current literature suggests that up to 40–80% of medical information is forgotten immediately and much of the information that is recalled is inaccurately retained [10]. One intervention that has been shown to be effective for improving information retention and understanding is the teach-back method [11–17]. Teach-back encourages healthcare providers to ask patients to explain, in their own words, the information that has been covered. If the patient is unable to recall or has difficulty understanding the information, the provider can identify specific misunderstandings/deficits and re-explain the concept. The benefits of teach-back have not yet previously been studied in the hepatitis B setting. Therefore, the aims of this study were to:

i) Assess baseline knowledge in people living with CHB;
ii) Evaluate the efficacy of the teach-back method for improving CHB knowledge, compared to a standard medical consultation.

## Method

### Trial design

This was a randomised, parallel group, two arm pilot study with an allocation ratio of 1:1.

### Patient cohort

In this randomised controlled trial, adults living with CHB referred to and attending the outpatient liver clinic at a Melbourne tertiary hospital were recruited between February and June 2017. Patients aged 18 years or older living with CHB and compensated liver disease were eligible for the study. Chronically infected was defined as HBsAg-positive for greater than 6 months. Recruitment of participants requiring an interpreter was piloted, but for logistic reasons the decision was made to focus on patients who were English-speaking by self-assessment. The results of the four participants who were interviewed with an interpreter were included in the final analysis. The barriers to interpreter access were cost and increased consultation time within a fixed recruitment period.

### Study procedure

All eligible patients were approached in the outpatient waiting rooms before their appointments and invited to participate in the study by the author (ST). Sociodemographic details were collected and the study questionnaire was administered to measure baseline knowledge. Participants were then randomised into the standard consult (control) or teach-back (intervention) group. A software-generated randomisation software (SAS V9.2) was used to create an allocation sequence to assign patients to the two study arms by the principal investigator (AT). The allocation sequence was concealed to the investigator recruiting patients and administering teach-back (ST). The CHB questionnaire was administered to participants immediately following their respective consultation and again at four weeks after the initial educational intervention (teach-back vs standard consult) over the phone. The main outcome measure was a combined knowledge score of the domains assessed – transmission, natural history, epidemiology and prevention and clinical management.

### Study questionnaire

A modified validated questionnaire (Additional files 1 and 2) was used in this study [7]. The first part of the questionnaire was a baseline survey to obtain sociodemographic details of participants (Additional file 1). The CHB questionnaire (Additional file 2) consisted of 23 true or false statements assessing hepatitis B knowledge scores across four domains: transmission (nine questions), natural history (five questions), epidemiology and prevention (five questions) and clinical management (four questions). The questionnaire was administered: i) at baseline; ii) immediately post-intervention to test early recall; and iii) 4 weeks post-intervention to test late recall. A knowledge score of ≥17.3 (75%) was considered as high knowledge and a score between 12.7–17.3 (55–75%) as intermediate level, as defined by previous study [7].

### Control and intervention

Participants in the control group attended their scheduled routine appointment with a gastroenterologist or gastroenterology advanced trainee registrar. A standard consult for a patient would involve reviewing their progress, recent investigations and if applicable, treatment regime. Participants in the intervention group received a once-off education session run by a trained investigator (ST) using the teach-back strategy. This session involved a discussion between the investigator and participant on the four domains of CHB – transmission, natural history, epidemiology and prevention and clinical management; supported by simple explanations and images using an educational resource specifically developed for people with low health literacy (The Hepatitis B Story [18]). Information was explained using plain language and medical jargon avoided. After covering each concept, the investigator would use teach-back to confirm patient understanding, for example: ‘To check that I have explained the information clearly, could you please tell me how hepatitis B is spread?’. If the answers were wrong or incomplete, the investigator would re-explain the concept and reassess until the participant understood the information.

### Statistical analysis

Overall knowledge was scored out of 23, based on answers to all of the questions across the four domains. One point was given for correct answers and zero for incorrect or a ‘do not know’ answer. Descriptive statistics were reported as mean ± standard deviation (SD) or median (interquartile range) for continuous data, and statistical summaries were reported as counts and percentages of participants with a positive response for categorical data.

Group equivalence was assessed by the Chi-square test and Fisher exact test on categorical variables and independent sample t-test on continuous variables. The intervention and control group knowledge scores were compared by paired t-test and analysis of variance (ANOVA) for normally distributed data and by Wilcoxon signed rank test for non-normally distributed data. Linear regression was conducted to detect associations between knowledge scores and sociodemographic characteristics. Variables that reached statistical significance (*p* ≤ 0.05) and those that showed a trend towards association with knowledge score (*p* ≤ 0.1 was accepted), were included in the multivariate model.

All analyses were performed on an intent-to-treat basis and included all enrolled participants. In all cases, comparisons were two-tailed and a *p*-value of ≤0.05 was considered statistically significant. Data was analysed using SPSS software version 23 (IBM, New York, 2015).

## Results

### Participant characteristics

Seventy participants were enrolled in this study within the fixed recruitment period, between February and June 2017 (control *n* = 32, teach-back *n* = 38, Table 1). No participants were excluded or lost at early recall, however 19 participants were lost at 1 month follow up (control: *n* = 8, intervention: *n* = 11). The majority of participants were born overseas, with 77% from Asia, 49% self-rated their English proficiency to be very good and 78% had tertiary qualifications. Mean time since diagnosis was 13.5 years (SD:10.9) and time since first consult was 7.2 years (SD:6.5) with one participant presenting for initial consult. Two thirds of participants had previously or are currently receiving antiviral treatment.

**Table 1:** Sociodemographic features of the study cohort

Sociodemographic characteristics		Control *n* = 32	Teach-back *n* = 38	Overall *n* = 70	*P*-value
Age (years)					
	Mean ± SD	42.5 ± 11.8	43.2 ± 13.2	42.9 ± 12.5	0.80
Gender *n*(%)					
	Male	18 (56)	21 (55)	39 (56)	0.93
	Female	14 (44)	17 (45)	31 (44)	
Region of Birth* *n*(%)					
	Australia	2 (6)	7 (18)	9 (13)	0.51
	South-East Asia^a^	16 (50)	17 (45)	33 (47)	
	North Asia^b^	10 (31)	10 (26)	20 (29)	
	Other^c^	4 (12)	4 (11)	8 (11)	
Time spent in Australia (years)					
	Mean ± SD	21.9 ± 10.4	23.6 ± 11.3	22.8 ± 10.9	0.51
Refugee Camp *n*(%)		5 (15)	5 (13)	10 (14)	0.99
Education level *n*(%)					
	Primary/Secondary	7 (22)	12 (32)	19 (27)	0.36
	Tertiary	25 (78)	26 (68)	51 (73)	
Self-rated English proficiency *n*(%)					
	Limited-Good	20 (62)	16 (42)	36 (51)	0.089
	Very Good	12 (38)	22 (58)	34 (49)	
Occupational status *n*(%)					
	Employed (full-time)	18 (56)	24 (63)	42 (60)	0.78
	Employed (part-time)	5 (16)	6 (16)	11 (16)	
	Other	9 (28)	8 (21)	17 (23)	
Time since diagnosis (years)					
	Mean ± SD	12.4 ± 8.7	14.4 ± 10.2	13.5 ± 10.9	0.39
Time since intial consultation at SVHM (years)					
	Mean ± SD	7.9 ± 7.2	6.5 ± 5.8	7.2 ± 6.5	0.38
Cirrhosis *n*(%)		1 (3)	1 (3)	2 (2.8)	0.99
Treatment *n*(%)					
	Current	21 (66)	17 (45)	38 (54)	0.15
	Previous	2 (6)	7 (18)	9 (13)	
	Never	9 (28)	14 (37)	23 (33)	

*Regions of birth were as follow:

^a^South-East Asia: Vietnam, Malaysia, Thailand, Indonesia, Philippines, Myanmar, Brunei, East Timor

^b^North Asia: China, Hong Kong, Taiwan, South Korea, Japan

^c^Other: France, Macedonia, India, Sudan, Mali, Pakistan, Papua New Guinea, Mauritius

### Baseline hepatitis B knowledge

The mean total pre-test knowledge score was 19.1 (2.4) out of 23. Fifty-five participants (79%) had high level hepatitis B knowledge, scoring ≥17.3. There was no difference in mean total knowledge at baseline between teach-back vs standard consultations arms (18.7 vs 19.4, *p* = 0.2). Sub-analysis of knowledge according to the four domains of HBV knowledge revealed good knowledge (mean scores): clinical management (75%; IQR: 75.100), natural history (100%; IQR: 80,100), transmission (78%; IQR: 67,100) and epidemiology and prevention (80%; IQR: 80,80).

Two questions answered correctly by all participants were ‘people with hepatitis B should tell their family members to get tested for hepatitis B’ and ‘hepatitis B causes liver damage’ (Table 2). Poorly answered questions included ‘washing hands can prevent hepatitis B’ (57%), ‘hepatitis B can be cured’ (61%) and ‘Asian people are more likely to have hepatitis B’ (66%). Forty-one participants (59%) were aware of all four transmission routes of hepatitis B (i.e. sexual and vertical transmission, sharing of injecting equipment, sharing toothbrushes and razor blades). Commonly misperceived modes of transmission included kissing (60%) and sharing eating utensils (57%).

**Table 2:** Baseline Knowledge - Proportion of participants correctly answered HBV questions

Questions	Control *n* = 32	Teach-back *n* = 38	Overall *n* = 70
Transmission routes	*N* (%)	*N* (%)	*N* (%)
By having unprotected sex with a person with hepatitis B (true)	26 (81)	30 (79)	56 (80)
Through mother to child at birth (true)	29 (91)	28 (74)	57 (81)
By touching a person with hepatitis B (false)	31 (97)	35 (92)	66 (94)
By kissing a person with hepatitis B (false)	21 (66)	21 (55)	42 (60)
By eating food prepared and cooked by a person with hepatitis B (false)	27 (84)	30 (79)	57 (81)
Through the air when a person with hepatitis B coughs or sneezes (false)	26 (81)	36 (95)	62 (89)
By sharing eating utensils (false)	17 (53)	23 (61)	40 (57)
By sharing toothbrushes or razor blades (true)	28 (88)	31 (82)	59 (84)
By sharing injecting equipments, e.g. Needles used in acupuncture, tattooing, body piercing or drug use (true)	32 (100)	37 (97)	69 (99)
Natural history			
Hepatitis B can cause liver damage (true)	32 (100)	38 (100)	70 (100)
Hepatitis B can cause liver cancer (true)	29 (91)	33 (87)	62 (89)
Most people infected with hepatitis b have no symptoms (true)	25 (78)	28 (74)	53 (76)
People with hepatitis B can be infected for life (true)	29 (91)	36 (95)	65 (93)
Alcohol further damages the liver for people with hepatitis B (true)	31 (97)	35 (92)	66 (94)
Epidemiology and prevention			
Asians are more likely to be infected with hepatitis B than other people (true)	22 (69)	24 (63)	46 (66)
There is a vaccination to prevent hepatitis B (true)	28 (88)	35 (92)	63 (90)
Washing hands before eating prevents getting hepatitis B (false)	19 (59)	21 (55)	40 (57)
People with hepatitis B should use condoms when having sex (true)	30 (94)	31 (82)	61 (87)
People with hepatitis B should tell their family members to get tested (true)	32 (100)	38 (100)	70 (100)
Clinical management			
Hepatitis B can be cured (false)	20 (63)	23 (61)	43 (61)
There are effective treatments for hepatitis B (true)	28 (88)	34 (90)	62 (89)
Hepatitis B can be cured by taking traditional Chinese medicine (false)	31 (97)	32 (84)	61 (87)
Healthy people with hepatitis B do not need regular check-up (false)	31 (97)	33 (87)	64 (91)

### Factors associated with baseline hepatitis B knowledge

Specific sociodemographic factors were correlated with baseline HBV knowledge. Linear regression analysis showed age ≤ 35 years and very good English proficiency were associated with better knowledge (Table 3). Multivariate analysis found that participants who had self-rated very good English proficiency (*p* = 0.016) and are currently receiving or have previously received antiviral treatment (*p* = 0.047), were independently associated with higher knowledge scores (Table 3).

**Table 3:** Regression analysis of association between HBV knowledge scores and sociodemographic features

Sociodemographic characteristics	*n* (%)	Mean Total knowledge score (SD)	Beta co-efficient (95% CI)	*P*-value	Beta co-efficient (95% CI)	*P*-value
			Univariable analysis		Multivariable analysis	
Gender						
Male	39 (56)	18.9 (2.4)				
Female	31 (44)	19.3 (2.5)	0.075 (−0.80, 1.52)	0.54		
Age						
> 35	49 (70)	18.7 (2.4)				
≤ 35	21 (30)	20 (2.3)	0.26 (0.13, 2.57)	0.031	1.06 (−1.53–2.27)	0.086
Birth Region						
Australia/Europe	11 (16)	18.9 (2.2)				
Asia	54 (77)	19.2 (2.4)	−0.051 (− 1.85, 1.23)	0.69		
Others	5 (7)	17.6 (3.4)	0.25 (−1.66, 4.28)	0.36		
Time spent in Australia						
≤ 15 years	21 (30)	19.2 (2.7)				
> 15 years	49 (70)	19.0 (2.3)	0.037 (−1.07, 1.45)	0.76		
Refugee Camp						
Yes	10 (14)	18.4 (2.2)				
No	60 (86)	19.1 (2.5)	0.078 (−1.11, 2.18)	0.52		
Education level						
Secondary and under	19 (27)	18.4 (2.2)				
Tertiary	51 (73)	19.3 (2.5)	0.15 (−0.49, 2.09)	0.22		
Self-rated English Proficiency						
Limited-good	36 (51)	18.3 (2.7)				
Very good	34 (49)	19.9 (1.8)	0.34 (0.52, 2.70)	0.005	1.36 (0.26–2.45)	0.016
Occupational Status						
Employed (full time)	42 (60)	18.8 (2.3)				
Other	28 (40)	19.5 (2.6)	−0.15(−1.91, 0.43)	0.21		
Time since diagnosis (years)						
≤ 10 years	41 (59)	19.0 (2.0)				
> 10 years	29 (41)	19.1 (2.7)	−0.008 (−1.21, 1.14)	0.95		
Time since initial consult at SVHM						
≤ 5 year	14 (20)	19.3 (1.9)				
> 5 year	56 (80)	19.0 (2.5)	0.048 (−1.16, 1.73)	0.69		
Treatment						
Never	23 (32)	18.5 (3.1)				
Current/Previous	47 (68)	19.3 (1.9)	0.17 (−0.35, 2.08)	0.16	1.16 (0.01–2.31)	0.047

### Effectiveness of teach-back

All participants randomised to teach-back received a dedicated hepatitis B education session in which the teach-back communication technique was used to reinforce and test knowledge retention. Median time taken to deliver a teach-back education session was 15 min (IQR: 12, 22). Hepatitis B education using teach-back was associated with improvement in hepatitis B knowledge scores. Participants in the teach-back group scored higher in the post-test compared to baseline (22.5 vs 18.7, *p* < 0.001). There was no improvement in hepatitis B knowledge associated with the standard clinical consultation (19.6 vs 19.4, *p* = 0.49). There were significant improvements across all four domains assessed within the teach-back group (Table 4).

**Table 4:** Comparison of knowledge scores according to domain

Domain		Mean ± SD	*P*-value	Mean ± SD	*P*-value
		Control *n* = 32		Teach-back *n* = 38	
Transmission	Baseline	7.4 ± 1.3	> 0.99	7.1 ± 1.6	< 0.001
	Early recall	7.4 ± 1.3		8.9 ± 0.4	
Natural history	Baseline	4.6 ± 0.7	0.03	4.5 ± 0.8	< 0.001
	Early recall	4.8 ± 0.5		5.0 ± 0.0	
Epidemiology & Prevention	Baseline	4.1 ± 0.6	> 0.99	3.9 ± 0.7	< 0.001
	Early recall	4.1 ± 0.6		4.8 ± 0.5	
Clinical Management	Baseline	3.4 ± 0.7	0.68	3.2 ± 0.9	< 0.001
	Early recall	3.3 ± 0.9		3.8 ± 0.5	

Following teach-back, all participants scored 100% in the natural history domain and correctly identified that sharing eating utensils was not a transmission route. A greater proportion of participants correctly answered questions identified to expose focal deficits in knowledge at baseline following teach-back: ‘washing hands before eating prevents hepatitis B’ (teachback: 95% vs baseline: 61%) (false) and kissing is a mode of transmission (teachback: 97% vs baseline: 55%) (false).

### Late recall

Fifty-one participants (control: *n* = 24, intervention: *n* = 27) successfully completed the 1-month follow-up. Nineteen participants were lost to follow up (control: *n* = 8, intervention: *n* = 11); there was a maximum of five attempts to contact participants in the week of their scheduled follow-up phone call. In the teach-back group, knowledge scores declined at 1-month follow-up compared to early recall (median knowledge score: 22 vs 23, *p* = 0.03). However, knowledge was found to remain higher when compared to baseline (median knowledge score: 22 vs 19, *p* < 0.001) (Table 5). The control group showed no significant change in knowledge scores both at 1-month follow-up compared to early recall (median knowledge score: 19 vs 20, *p* = 0.72) and at 1-month compared to baseline (median knowledge score 19 vs 19.5, *p* = 0.65).

**Table 5:** Comparison of knowledge scores at 1-month post-intervention

	Median (IQR) Control *n* = 24	*P*-value	Median (IQR) Teach-back *n* = 27	*P*-value
Baseline	19.5 (18.25,21)	0.65	19 (17.75,20)	< 0.001
Late recall	19 (18,21.75)		22 (21,23)	
Early recall	20 (18,21)	0.72	23 (22,23)	0.03
Late recall	19 (18,21.75)		22 (21, 23)	

## Discussion

This is the first study to test the effectiveness of patient education using the teach-back communication technique in people living with CHB. We have demonstrated that a short education session using this simple communication tool is associated with improvement in levels of hepatitis B knowledge.

Teach-back was effective in improving knowledge across all domains assessed. Its use was also shown to enhance information retention at 1-month post-intervention. Though there was a decline in knowledge scores between early and late recall, there was a continual improvement when compared to baseline. A regression in knowledge score at follow-up may not necessarily reflect the effect of teach-back on information maintenance but rather the nature of the educational session. A large number of concepts were covered in the one session and despite this, participants continued to demonstrate a good degree of information retention.

A study assessing how doctors used teach-back revealed a selective approach based on education level, ethnic background and age [19]. A downfall to this approach is that patients who do not fit these criteria, but may benefit from teach-back, are being overlooked. Our study cohort, in some respects, represents a privileged population; with higher education and English-proficiency levels, a mean time of 7.2 years of follow up in clinic and two thirds previously or currently on treatment. Though teach-back has been mostly studied in cohorts with low health literacy; our study, which has demonstrated an significant improvement in disease understanding within a privileged cohort, would support the routine use of teach-back with all participants regardless of sociodemographic profile. Our findings also validate the recommended ‘universal precautions’ approach to health literacy, that is health providers should assume the default to be that everyone has some difficulty in accessing and understanding health information [20]. An Australian study reported that when solely based on education levels, more than 10% of patients were incorrectly assumed to have adequate/limited health literacy [21]. Health literacy extends beyond an individual’s literacy and numeracy skills. It encompasses cognitive and social skills which influences an individual’s capacity to promote and maintain good health by understanding and effectively applying health information [22].

Teach-back is a simple communication tool with a small evidence base for its successful use and our data adds to this existing literature that supports its use. Teach-back has been studied in a range of contexts such as discharge from the emergency department [15] and in chronic diseases such as diabetes [13] and asthma/COPD [17]. Across these studies, teach-back consistently showed improvement in disease-specific knowledge. Furthermore, a study comparing a brief verbal and written educational intervention with teach-back in educating hospitalised patients with asthma/COPD on correct respiratory inhaler techniques; found teach-back to be more effective in reducing inhaler misuse and number of hospital-related events [17].

Overall our study cohort’s baseline knowledge was high compared to previous studies assessing hepatitis B knowledge amongst patients living with CHB [7–9]. Notable differences between our study cohort and past studies, were exclusion of participants requiring interpreters, higher level of education, long-term clinic patients and higher self-assessed English proficiency. Similar to previous studies, English proficiency was found to be associated with higher levels of hepatitis B knowledge [7]. As such, exclusion of non-English speaking patients within our study would be a large contributing factor to our study cohort’s higher baseline knowledge. Our study also showed that participants who currently or have previously received antiviral therapy had higher knowledge. This may be explained by the fact that individuals who are on treatment may have had more information provided regarding indications for treatment and aim of treatment. They may also have more frequent follow-ups and therefore opportunities to engage them in health services and to address queries.

The deficits in knowledge identified concerned transmission and whether hepatitis B is curable. Understanding transmission routes is important to prevent ongoing transmission to susceptible contacts, to promote screening and vaccination among close contacts and also to improve quality of life by addressing misconceptions of transmission routes that contribute to the stigma of hepatitis B and cause social withdrawal [23]. Patients who understand that the role of antiviral therapy is not to cure but rather to suppress the virus may be more likely to appreciate the importance of adherence to medication and the need for ongoing long-term virological monitoring and engagement with HCC surveillance, all important management aspects of hepatitis B.

### Limitations of this study

There are a number of limitations to this study. Though a large proportion of people living with CHB are from CALD communities, our study cohort did not include non-English-speaking patients. Future studies should evaluate hepatitis B knowledge and the use of teach-back in patients with limited health literacy, particularly in CALD communities. This is a simple education tool that would be feasible to apply with non-English speaking individuals with an interpreter. Furthermore, it would be interesting to evaluate this tool in patients newly diagnosed with hepatitis B. Engaging patients early in their disease course may increase adherence to important management aspects such as transmission precautions, monitoring and surveillance to mitigate the long-term consequences of hepatitis B. In this study, teach-back use was explored as a once-off education session; more research should explore its repeated use; the use of focussed teach-back over multiple appointments would also decrease time requirements. Finally, it will be important to evaluate how increased disease-related knowledge can translate into better health outcomes.

## Conclusion

This is the first randomised controlled study to evaluate the use of teach-back to improve understanding among people living with CHB. There was an overall high level of baseline disease knowledge in this cohort, however focal deficits in knowledge were identified. The use of teach-back was associated with significant improvement in knowledge and information retention. It is a simple communication tool that could be incorporated in standard clinical consults. Future studies should evaluate the efficacy of teach-back for improving hepatitis B knowledge in populations at risk for poor health literacy, in particular CALD communities.

## Supplementary information

**Additional file 1.** Baseline survey to obtain sociodemographic information.

**Additional file 2.** A modified validated questionnaire to assess knowledge on hepatitis B relating to the following domains: transmission, natural history, epidemiology and prevention and clinical management.

## Data Availability

The study protocol, study questionnaire and datasets used and analysed during the current study are available from the corresponding author on reasonable request.

## Abbrieviations

CALD: Culturally and linguistically diverse
CHB: Chronic hepatitis B
HBV: Hepatitis B
HCC: Hepatocellular carcinoma
