# Supplementary material for: ‘Teach-back’ is a simple communication tool that improves disease knowledge in people with chronic hepatitis B – a pilot randomized controlled study
Source: BMC Public Health. 2019 Oct 23;19:1355. doi: 10.1186/s12889-019-7658-4 (PMC6813056; doi:10.1186/s12889-019-7658-4)
Supplement: Supplementary file 1 — Additional file 1. Baseline survey to obtain sociodemographic information. [file 12889_2019_7658_MOESM1_ESM.docx]

**Additional File 1.** Baseline Survey

**BASELINE SURVEY**

1. Name
2. Sex Male Female
3. Date of Birth / /
4. Are you born in Australia? Yes No
5. If no, please list your country of birth

1. How long have you lived in Australia?
2. What is your ethnicity?
3. Have you lived in a refugee camp? Yes No
4. What is your highest level of education?

Primary ☐

Secondary ☐

Tertiary ☐

1. Do you use an interpreter during your consultations?

Trained ☐

Family/Friend ☐

None ☐

1. If so, which language?
2. How you would rate your level of English language proficiency?

No English ☐

Limited ☐

Good ☐

Very good ☐

Not specified ☐

1. What is your occupational status?

Employed full time (including self-employed) ☐

Employed part time (including self-employed) ☐

Student ☐

Unable to work due to illness ☐

Home duties ☐

Retired ☐

Currently looking for work ☐

Not looking for work ☐

Not specified ☐
