# Supplementary material for: ‘Teach-back’ is a simple communication tool that improves disease knowledge in people with chronic hepatitis B – a pilot randomized controlled study
Source: BMC Public Health. 2019 Oct 23;19:1355. doi: 10.1186/s12889-019-7658-4 (PMC6813056; doi:10.1186/s12889-019-7658-4)
Supplement: Supplementary file 2 — Additional file 2. A modified validated questionnaire to assess knowledge on hepatitis B relating to the following domains: transmission, natural history, epidemiology and prevention and clinical management. [file 12889_2019_7658_MOESM2_ESM.docx]

**Additional File 2.** HBV Questionnaire

TRANSMISSION ROUTES

***This section looks at how hepatitis B is spread. Please tick the following statements that are true.***

☐ By having unprotected sex with a person with hepatitis B

☐ Through mother to child at birth

☐ By touching a person with hepatitis B

☐ By kissing a person with hepatitis B

☐ By eating food prepared and cooked by a person with hepatitis B

☐ Through the air when a person with hepatitis B coughs or sneezes

☐ By sharing eating utensils

☐ By sharing toothbrushes or razor blades

☐ By sharing injecting equipment, for example, needles used in acupuncture, tattooing, body piercing or drug use

NATURAL HISTORY

***This section looks at the symptoms and complications of hepatitis B infection. Please tick the following statements that are true.***

☐ Hepatitis B can cause liver damage

☐ Hepatitis B can cause liver cancer

☐ Most people infected with hepatitis B have no symptoms

☐ People with hepatitis B can be infected for life

☐ Alcohol can further damage the liver for people with hepatitis B

EPIDEMIOLOGY AND PREVENTION

***This section looks at ways to prevent the spread of hepatitis B. Please tick the following statements that are true.***

☐ Asians are more likely to be infected with hepatitis B than other people

☐ There is a vaccination to prevent hepatitis B

☐ Washing hands before eating prevents getting hepatitis B

☐ People with hepatitis B should use condoms when having sex

☐ People with hepatitis B should tell their family members to get tested for hepatitis B

CLINICAL MANAGEMENT

***This section looks at how hepatitis B can be managed. Please tick the following statements that are true.***

☐ Hepatitis B can be cured

☐ There are effective treatments for hepatitis B

☐ Hepatitis B can be cured by taking traditional Chinese medicine

☐ Healthy people with hepatitis B do not need regular check-ups
